# Variations in Sense of Place Across Immigrant Status and Gender in Hamilton, Ontario; Saskatoon, Saskatchewan; and, Charlottetown, Prince Edward Island, Canada

**DOI:** 10.1007/s11205-014-0636-4

**Published:** 2014-04-26

**Authors:** Melissa Gallina, Allison Williams

**Affiliations:** School of Geography and Earth Sciences, McMaster University, 1280 Main Street West, Hamilton, ON L8S 4K1 Canada

**Keywords:** Sense of place, Immigrants, Canada, Small-to-medium sized cities, Gender

## Abstract

Past research in Hamilton, Ontario has found that age and longevity of residence are positively associated with evaluations of sense of place (SoP); further, evaluations of SoP between immigrants and Canadian-born individuals have shown no clear pattern (Williams et al. 2010; Williams and Kitchen 2012). This paper builds on this work by further examining evaluations of SoP among both immigrants and Canadian-born residents and across gender in Hamilton, while expanding the study to two other small-to-medium sized cities: Saskatoon, Saskatchewan and, Charlottetown, Prince Edward Island. This paper has two objectives: (1) to establish measures of SoP across immigrant status and gender in Hamilton, Saskatoon, and Charlottetown; and, (2) to determine how SoP varies according to immigrant status, length of residence in Canada, age, income, and neighbourhood length of residence across the three city sites. Telephone survey data (n = 1,132) was used to compare evaluations of SoP across various groups and to construct an ordered logistic regression model for SoP. Results suggest that immigrants tended to rate their SoP lower than their Canadian-born counterparts. Hamilton residents were found to rate their SoP lowest, followed by Saskatoon residents and, finally, Charlottetown residents. Younger individuals, those with lower income levels, and those with shorter neighbourhood residency in the cities concerned were more likely to have lower evaluations of SoP. This research suggests that greater attention is needed to nurture immigrants’ connection with their new home.

## Introduction

Canada remains a popular destination for immigrants (CIC 2011), with the majority settling in Canada’s three largest cities: Toronto, Vancouver, and Montreal (Frideres 2006; Radford 2007). However, there is an increasing number of immigrants settling in smaller Canadian cities, which suggests a need to explore immigrant experiences in these smaller centres (Frideres 2006; Radford 2007). Sense of place (SoP) has been defined as “the attitudes and feelings that individuals and groups hold *vis*-*à*-*vis* the geographical areas in which they live. It further commonly suggests intimate, personal and emotional relationships between self and place” (Wylie 2009 676). As such, SoP will be used to evaluate the immigrant experience in three small-to-medium sized cities. Information about SoP provides insight into the emotional attachments immigrants have established with their new home, providing a rationale for this research. It is reasoned that a strong SoP may assist in the adaptation process of immigrants. Additionally, individual perceptions of place have been related to health and wellbeing (DeMiglio and Williams 2008; Williams and Kitchen 2012), thus providing important information about the immigrant experience.

This paper has two objectives: (1) to establish measures of SoP across immigrant status and gender in Hamilton, Saskatoon, and Charlottetown; and, (2) to determine how SoP varies according to immigrant status, length of residence in Canada, age, income, and neighbourhood length of residence across the three city sites. As such, this paper adds to current literature on SoP in the three cities. This paper builds on previous work by further examining evaluations of SoP among both immigrants and Canadian-born residents and across gender in Hamilton, while expanding the study to two other small-to-medium sized cities: Saskatoon, Saskatchewan and, Charlottetown, Prince Edward Island. The rationale for expanding the study to Saskatoon and Charlottetown is to determine if the results found in previous studies conducted in Hamilton apply in other Canadian cities. With this addition, the study contains cities from each of the three major geographical regions of Canada: Western, Central, and Atlantic. It is noted that the cities chosen constitute a convenience sample, and not a random sample of cities within each region.

This paper begins with a review of the literature surrounding: immigration in Canada; contextual information on each of Hamilton, Saskatoon, and Charlottetown; and, neighbourhood SoP in the three city sites. Next, the data and methods of analysis are discussed. Analysis was conducted using an established SoP formula (Williams et al. 2010) and ordered logistic regression modelling. Results are presented using graphs and tables. A discussion of results is provided, which acknowledges the limitations of this study. Finally, this paper presents a conclusion, which includes policy recommendations to increase immigrant SoP.

## Literature Review

### Immigration in Canada

Canada is a country that was built by immigrants and continues to be a popular immigrant destination today (CIC 2011). Each year, Canada absorbs approximately 250,000 permanent residents and 200,000 temporary foreign workers and international students (CIC 2011). In recent years, Canada has had a per capita immigration rate of 0.8 %, representing one of the highest rates in the world (CIC 2011). Approximately 75 % of all immigrants to Canada reside in Toronto, Vancouver, and Montreal (Frideres 2006; Radford 2007). In comparison, just over 25 % of Canadian-born individuals reside in one of Canada’s three largest cities (Frideres 2006). A goal of the Canadian immigration policy is to encourage economic growth in smaller centres (Green 2003; Green and Green 1999). It is suggested that immigrants provide the necessary labour and skills that these areas require to grow (Green 2003; Green and Green 1999). As mentioned above, a greater number of immigrants are settling in smaller Canadian cities, providing the rationale to study the immigrant experience in these sites (Frideres 2006; Radford 2007). For the purpose of this study, immigrant status is defined by location of birth; where immigrants are defined as those not born in Canada.

### Site Context

Hamilton, Ontario is located in central Canada, with industry focused on services and manufacturing (Statistics Canada 2007b). The population of Hamilton is 519,949 (Statistics Canada 2012b), including 3,296 immigrants (CIC 2011). Saskatoon, Saskatchewan, located in western Canada, has a population of 234,000 (City of Saskatoon 2011), including 3,796 immigrants (CIC 2011). Farming and mining are the primary industries in Saskatoon (City of Saskatoon 2011). Located in eastern Canada, Charlottetown’s industry is focused on services and retail trade (Statistics Canada 2007a). The population of Charlottetown is 34,562 (Statistics Canada 2012a), 1,665 of which are immigrants (CIC 2011).

### Sense of Place

SoP has been related to a number of similar constructs, including: place attachment, sense of community, community satisfaction, and place identity. This research focuses on SoP, which reflects the experiences and perceptions of the residents themselves (Williams et al. 2010). In comparison, other place-related concepts, such as place identity, are informed by external views and stereotypes (Williams et al. 2010). Place attachment, an environmental psychology measure, focuses more heavily on psychological understandings, and less on place-based understandings (Williams et al. 2010). Of the aforementioned terms, SoP describes the purest geographical understanding of the relationship between people and place (Williams et al. 2010).

A select number of studies have examined SoP in Hamilton, Saskatoon, and Charlottetown. A study conducted by Williams et al. (2010) investigated the influence of neighbourhood socio-demographic characteristics on evaluations of SoP in Hamilton. SoP was stronger among residents of a higher income neighbourhood than residents of a lower income neighbourhood (Williams et al. 2010). Both neighbourhood length of residence and age were significant predictors of SoP, indicating an increased SoP over time (Williams et al. 2010). Gender and household income were not found to influence evaluations of SoP (Williams et al. 2010). Interestingly, immigrant respondents were found to have a higher SoP than Canadian-born respondents (Williams et al. 2010). In 2012, Williams and Kitchen conducted a follow-up study of SoP in Hamilton. Again, it was found that higher SoP was related to: higher socioeconomic status; older age; and, long-term neighbourhood residency (Williams and Kitchen 2012). Confirming previous results, gender was not a significant predictor of SoP (Williams and Kitchen 2012). In contrast to previous results, immigrant status was found to influence SoP, with immigrants having lower evaluations of SoP compared to Canadian-born individuals (Williams and Kitchen 2012).

Williams et al. (2008) also examined SoP in Saskatoon between 2001 and 2004. Evaluations of SoP were based on four variables: “(1) feeling part of the neighbourhood; (2) comfort in participating in neighbourhood projects; (3) calling on neighbours in a crisis; and, (4) volunteering for organizations” (Williams et al. 2008 17). Long-term neighbourhood residency; participating in volunteerism; and, perceptions of neighbourhood friendliness were found to be significant positive influences on SoP (Williams et al. 2008). Overall, SoP was found to be higher in 2001 than in 2004 (Williams et al. 2008). There is comparatively little research on SoP in Charlottetown, however, it has been suggested that island communities foster a ‘unique’ SoP due to their physical geography (Baldacchino 2005).

## Methods

### Data Summary

Data for this project was obtained from a telephone survey (n = 1,132) conducted in the summer of 2012, as part of a larger quality of life study funded by the Social Sciences and Humanities Research Council of Canada, SSHRC Immigration and the Metropolis (Table 1). The quality of life survey addressed a number of topics, including: health, social capital and, environmental problems. In addition, the survey contained a 16-item SoP scale. As shown in Table 1, approximately 75 % of survey respondents were born in Canada, while 25 % are immigrants. Survey respondents are split between both genders. The greatest proportion of respondents resides in Hamilton, followed by Saskatoon and Charlottetown, reflecting the relative size of each of the cities. Sample size, for both the number of households and the size of the immigrant population, were informed by the 2006 census. Households were randomly selected in each of the three cities. All immigrants, regardless of period of residency, were targeted for the survey. As part of the larger study, a series of tests comparing the sample to the 2006 census were carried out to verify the accuracy of the overall survey sample (Williams et al. under review). The comparisons indicated a high degree of accuracy with respect to various socio-demographic characteristics for immigrants (Williams et al. under review).

**Table 1:** Data summary

(n = 1,132)	Immigrant status				Gender				Total	
	Canadian-born		Immigrant		Male		Female			
	(n)	(%)	(n)	(%)	(n)	(%)	(n)	(%)	(n)	(%)
Charlottetown	191	85.65	32	14.35	99	44.39	124	55.61	223	19.70
Hamilton	351	67.11	172	32.89	226	43.21	297	56.79	523	46.20
Saskatoon	305	79.02	81	20.98	203	52.59	183	47.41	386	34.10
Total	847	74.82	285	25.18	528	46.64	604	53.36	1,132	100.00

The SoP scale, established by Williams et al. (2010), focuses on four areas: neighbourhood rootedness; neighbourhood sentiment; neighbours; and, environment and health, each of which is made up of four questions (See Table 2). Data for each of the sixteen items was collected using a five-point likert scale and was later coded to represent values between one and five (Williams et al. 2010). Values of one represent the most positive responses and values of five represent the most negative responses (Williams et al. 2010). As an exception, three of the environment and health questions (D7, D8 and D9) were reverse coded, as these questions refer to negative influences of SoP (Williams et al. 2010). This was done in order to ensure consistency with the other questions, which reflect positive influences on SoP (Williams et al. 2010).

**Table 2:** 16-item sense of place scale (Williams et al. 2010)

*The following statements and questions have to do with your feelings about your neighbourhood. For each, indicate the degree to which you agree or disagree.*				
**D1. My neighbourhood means a great deal to me.**				
1 (Strongly agree)	2 (Agree)	3 (Neutral)	4 (Disagree)	5 (Strongly disagree)
**D2. There’s no other neighbourhood I would rather live.**				
1 (Strongly agree)	2 (Agree)	3 (Neutral)	4 (Disagree)	5 (Strongly disagree)
**D3. I feel at home in my neighbourhood.**				
1 (Strongly agree)	2 (Agree)	3 (Neutral)	4 (Disagree)	5 (Strongly disagree)
**D4. There are people in my neighbourhood who I think of as close friends.**				
1 (Strongly agree)	2 (Agree)	3 (Neutral)	4 (Disagree)	5 (Strongly disagree)
**D5. I would like to stay in my neighbourhood as long as my health allows me to do so.**				
1 (Strongly agree)	2 (Agree)	3 (Neutral)	4 (Disagree)	5 (Strongly disagree)
**D6. Green space availability in my neighbourhood positively influences my health.**				
1 (Strongly agree)	2 (Agree)	3 (Neutral)	4 (Disagree)	5 (Strongly disagree)
**D7. Environmental problems in my neighbourhood (e.g. air pollution, run-down buildings) negatively influence my health.**				
1 (Strongly agree)	2 (Agree)	3 (Neutral)	4 (Disagree)	5 (Strongly disagree)
**D8. Social problems in my neighbourhood (e.g. racism, violence) negatively influence my health.**				
1 (Strongly agree)	2 (Agree)	3 (Neutral)	4 (Disagree)	5 (Strongly disagree)
**D9. The personal safety of myself and my family in my neighbourhood negatively affects my health**				
1 (Strongly agree)	2 (Agree)	3 (Neutral)	4 (Disagree)	5 (Strongly disagree)
*How true are the following two statements?*				
**D10. I know many of my neighbours on a first name basis.**				
1 (Very true)	2 (Fairly true)	3 (Neutral)	4 (Not very true)	5 (Not at all true)
**D11. If I were to live somewhere else, it would be difficult to move away from my neighbourhood.**				
1 (Very true)	2 (Fairly true)	3 (Neutral)	4 (Not very true)	5 (Not at all true)
*I would like to ask you several more questions on how you feel about your neighbourhood.*				
**D13. How rooted do you feel in your neighbourhood?**				
1 (Very rooted)	2 (Fairly rooted)	3 (Neutral)	4 (Not very rooted)	5 (Not at all rooted)
**D14. How connected do you feel to your neighbourhood?**				
1 (Very connected)	2 (Fairly connected)	3 (Neutral)	4 (Not very connected)	5 (Not at all connected)
**D15. How much do you like your neighbourhood?**				
1 (A great deal)	2 (A fair amount)	3 (Neutral)	4 (Not very much)	5 (Not at all)
**D16. How often do you participate in social activities with your neighbours (e.g. barbeques, coffee dates, etc.)**				
1 (All the time)	2 (Often)	3 (Sometimes)	4 (Hardly ever)	5 (Never)
**D17. If you had to leave your neighbourhood, how many of your neighbours would you miss?**				
1 (Many of them)	2 (Some of them)	3 (Neutral)	4 (Hardly any of them)	5 (None of them)

*Factors*

Neighbourhood rootedness = D2 + D13 + D5 + D11

Neighbourhood sentiment = D1 + D3 + D14 + D15

Neighbours = D10 + D16 + D4 + D17

Environment and health = D6 + D7 + D8 + D9

### Analysis

Analysis for this paper took place in two phases. The first phase of the analysis aimed to determine if there was a significant difference in evaluations of SoP based on city of residence, immigrant status, and gender. Data from each of the three sites was imported into SAS and merged into a single data file. Any records with a missing response to one of the sixteen SoP questions were eliminated from the analysis. Individual evaluations of neighbourhood SoP were calculated using an equation (below) established by Williams et al. (2010). The equation is the result of a principal components analysis using 46 variables that have been found to influence neighbourhood SoP (Williams et al. 2010). As shown in Table 2, the 16-item survey is divided into four factors with each factor containing four questions (Williams et al. 2010).

$$SoP = \left[ {\frac{(20 - \varSigma Factor1) + (20 - \varSigma Factor2) + (20 - \varSigma Factor3) + (20 - \varSigma Factor4)}{64}} \right] \times 100$$

The sum of each factor can range from a low of four, which indicates a positive evaluation of SoP, to a high of 20, which indicates a weak SoP (Williams and Kitchen 2012). In order to prevent confusion, the values were reversed by subtracting the sum from 20 (Williams and Kitchen 2012). In doing so, the possible outcomes range from 0 (lowest possible SoP) to 16 (highest possible SoP; Williams and Kitchen 2012). Finally, the four sums are divided by 64 (the highest possible score) to obtain a percentage (Williams and Kitchen 2012). Values close to 100 represent a strong SoP (Williams and Kitchen 2012). An example is provided below using the data from the first Charlottetown respondent.

$$SoP = \left[ {\frac{{\left( {20 - 10} \right) + \left( {20 - 7} \right) + \left( {20 - 13} \right) + \left( {20 - 8} \right)}}{64}} \right] \times 100 = \left[ {\frac{{\left( {10 + 13 + 7 + 12} \right)}}{64}} \right] \times 100 = 65.6$$

SoP was compared (1) between immigrants and Canadian-born individuals; (2) between the three cities; and, (3) between males and females. An average value of SoP was calculated for each of these groups. In order to determine if evaluations of SoP differ significantly, 95 % confidence intervals were calculated for the mean SoP. An assumption of normality was made for the dataset, since both skewness and kurtosis were close to zero (Delwiche and Slaughter 2008).

The second phase of analysis involved the creation of an ordered logistic regression model to predict SoP using the following independent variables: immigrant status, city of residence, length of residence in Canada, income class, age category and neighbourhood length of residence. In order to conduct an ordered logistic regression, four categories of SoP were created. A standard Z-score was calculated for each individual based on their raw SoP score. The resulting Z-scores were used to create four SoP categories (Table 3).

**Table 3:** Z-scores for SoP Categories

Z-score	Sense of place
Z ≥ 1	High
<1 Z > 0	Above average
<0 Z > −1	Below average
Z ≤ −1	Low

In order to further investigate factors affecting SoP within each of the cities, attempts were made to stratify the sample and create separate models for each of the three cities. The models for Saskatoon and Charlottetown were not statistically significant, due to the small sample size in these two cities.

## Results

**Fig. 1:**
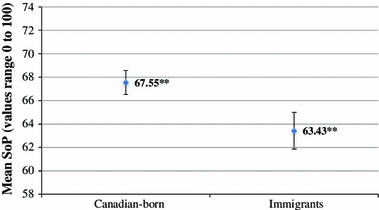
Sense of place by immigrant status, **statistically significant difference at the 95% confidence level

**Fig. 2:**
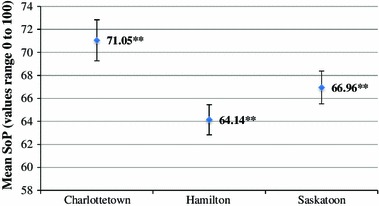
Sense of place by city of residence, **statistically significant difference at the 95% confidence level

**Fig. 3:**
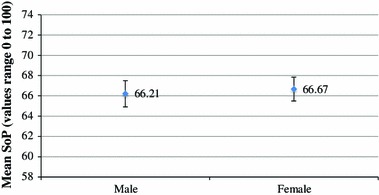
Sense of place by gender, no statistically significant difference at the 95% confidence level

Results will be presented in each of the two phases addressed in the methods section. During phase one, confidence intervals were used to determine if there was a significant difference in evaluations of SoP across groups. The scatterplots below (Figs. 1–3) display the mean SoP, confidence limits (95 %) and error bars. First, SoP was compared between immigrants and Canadian-born individuals across all three sites. As shown in Fig. 1 (below), Canadian-born individuals had an average SoP of 67.55 CI (66.53–68.57) and immigrants had an average SoP of 63.43 CI (61.86–65.00). At a 95 % level of confidence, the means for immigrants and Canadian-born individuals are significantly different.

Next, SoP was compared between the three sites. As shown in Fig. 2 (below), respondents from Charlottetown had an average SoP of 71.05 CI (69.26–72.84), respondents from Hamilton had an average SoP of 64.14 CI (62.83–65.45), and respondents from Saskatoon had an average SoP of 66.96 CI (65.53–68.39). At a 95 % confidence level, the mean for Charlottetown was significantly different from that of both Hamilton and Saskatoon. The means for Hamilton and Saskatoon are also significantly different at a 95 % confidence level; however, this should be interpreted with caution as the confidence intervals are very close to overlapping.

Finally, SoP was compared across gender. As shown in Fig. 3, males and females report very similar levels of SoP, 66.21 CI (64.93–67.49) and 66.67 CI (65.51–67.84) respectively. Therefore, there is not a significant difference in evaluations of SoP across gender.

**Fig. 4:**
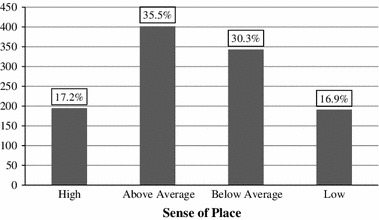
Descriptive statistics, sense of place

In phase two, ordered logistic regression was used to identify significant predictors of SoP for the whole sample. Figure 4 displays the distribution of respondents (n = 1,132) according to the four categories of SoP. The majority of respondents reported an “above average” SoP (35.5 %) or a “below average” SoP (30.3 %). A smaller proportion of respondents reported a “high” or “low” SoP (17.2 and 16.9 % respectively).

Significant predictors of SoP (*p* < 0.05) were found to be: city of residence; income; age; and, neighbourhood length of residence, all of which are highlighted in Table 4. Compared to residents of Saskatoon, residents of Hamilton are 1.6 times more likely to rate their SoP as “low” (closer to a value of 1 for the dependent variable). Residents of Charlottetown are 0.8 times less likely to rate their SoP as “low” when compared to those living in Saskatoon (close to statistical significance). Compared to the highest income class ($100,000+), individuals in the lower income categories are 1.4–2.2 times more likely to rate their SoP as “low”. Individuals under age 65 are 1.7–3.4 more likely to rate their SoP as “low” compared to those in the highest income category. Finally, those with shorter lengths of neighbourhood residence are 1.7–3.3 times more likely to rate their SoP as “low” compared to those living in their neighbourhood for 10 or more years.

**Table 4:** Ordered logistic regression model, SoP (n = 1,132)

Variable	Odds ratio	95 % CI
Immigrant status (ref = Immigrant)		
Canadian-born	0.836	0.626–1.116
City of residence (ref = Saskatoon)		
Charlottetown	0.752*	0.552–1.025
Hamilton	1.569**	1.212–2.032
Length of residence in Canada (ref = 10 + years)		
<1 year	0.752	0.267–2.117
1–5 year(s)	0.908	0.456–1.809
6–10 years	0.794	0.343–1.840
Income (ref = $100,000+)		
<$20,000	2.230**	1.522-3.268
$20,000–$39,999	1.867**	1.283–2.716
$40,000–$59,999	1.710**	1.212–2.413
$60,000–$79,999	1.408*	0.980–2.022
$80,000–$99,999	1.580**	1.050–2.380
Age (ref = 75+)		
18–24	2.196**	1.252–3.853
25–34	3.441**	2.076–5.703
35–44	2.281**	1.418–3.670
45–54	2.638**	1.693–4.111
55–64	1.689**	1.078–2.645
65–74	0.956	0.585–1.562
Neighbourhood length of residence (ref = 10 + years)		
<1 year	3.345**	2.001–5.590
1–5 year(s)	2.231**	1.644–3.029
6–10 years	1.748**	1.295–2.360

** Statistically significant at *p* < 0.05; * Close to statistical significance at *p* < 0.05

## Discussion

Four variables were found to be significant predictors of SoP: income; age; neighbourhood length of residence; and, city of residence. Individuals with lower household incomes were more likely to have lower evaluations of SoP. Past research has not found income to be a significant predictor of SoP (Williams et al. 2010). However, past studies have found that higher socioeconomic status neighbourhoods have higher evaluations of SoP (Williams et al. 2010; Williams and Kitchen 2012). It is reasoned that individuals with higher incomes have greater residential mobility and can choose to live in neighbourhoods that are conducive to SoP (Williams et al. 2010). Confirming past research, this research suggests that lower evaluations of SoP are more common amongst younger age groups and individuals with shorter lengths of neighbourhood residency (Williams et al. 2010; Williams and Kitchen 2012). As an individual spends more time in a neighbourhood and forms stronger connections with neighbours and place, they are likely to develop a stronger SoP (Williams et al. 2010; Williams and Kitchen 2012). Agreeing with past work, gender was not found to influence evaluations of SoP (Williams et al. 2010; Williams and Kitchen 2012).

When comparing evaluations of SoP by city, residents of Hamilton and Saskatoon had significantly lower evaluations of SoP compared to residents of Charlottetown (Fig. 2). Attempts to investigate within-city factors using quantitative analysis were made, however, the sample sizes in Saskatoon and Charlottetown were too small to produce a stratified model. Nevertheless, anecdotal evidence suggests that immigrant service providers in Charlottetown go beyond traditional measures in order to establish relationships between immigrants and Canadian-born individuals^1^, which may strengthen SoP. Past research has shown that settlement and other services are more personalized in smaller cities due to lower demand for services (Frideres 2006). In contrast, larger cities tend to employ ‘one-size-fits-all’ programs due to cost constraints (Frideres 2006). Both Saskatoon and Hamilton are substantially larger that Charlottetown, which may negatively impact the delivery of immigrant services and the establishment of SoP. In addition, the strong SoP observed among Charlottetown residents may be evidence of the Island’s ‘unique’ SoP, as mentioned above.

Immigrant status was not found to be a significant predictor of SoP; however, phase one indicated that immigrants have lower evaluations of SoP compared to Canadian-born individuals (Fig. 1). The lower levels of SoP found amongst immigrant respondents may be explained by a ‘disruption’ of SoP. Upon immigration to Canada, individuals must form new connections with people and place, often resulting in stress (Brown and Perkins 1992; Ng 1998). It should also be noted that the immigrant population in Canada has both a younger age distribution and a lower median income than the Canadian-born population (Fougère et al. 2004; Heisz and McLeod 2004; Sharpe 2011). As discussed above, both age and income are significant predictors of SoP. These factors, rather than some innate characteristic of being an immigrant, may explain the lower SoP found amongst immigrants.

Limitations of this project include the small sample size of immigrants in each city. The small sample size is most noticeable in Charlottetown, where there were only 32 immigrants in the sample. A second limitation involves the nature of the SoP formula. Individuals who did not answer all sixteen questions were excluded from the analysis. This could introduce a bias towards those who answered all the questions.

## Conclusion

More research is needed to examine the immigrant experience in smaller Canadian cities due to increased immigrant settlement in these areas. This study has evaluated immigrant SoP in three such cities: Hamilton, Saskatoon, and Charlottetown. Using the established SoP formula, evaluations of SoP were compared across immigrant status, city of residence, and gender. Immigrants were found to have lower evaluations of SoP compared to their Canadian-born counterparts. In terms of city of residence, Hamilton residents reported the lowest evaluations of SoP, followed by Saskatoon and Charlottetown residents. Ordered logistic regression identified four significant predictors of SoP across the entire sample: city of residence; income; age; and, neighbourhood length of residence.

This research proposes a number of recommendations to increase immigrant SoP. The recommendations proposed are relevant in small-to-medium-sized cities across Canada, since the cities are representative of three major Canadian regions. Recognizing the intimate relationship between income and employment, it is suggested that increased recognition of foreign skills and credentials could positively influence immigrant SoP. Immigrants face a number of barriers to employment such as: low English language proficiency; lack of Canadian work experience; devaluation of foreign work experience; and, the lack of recognition of foreign training and education credentials (Galarneau and Morissette 2009; Tran 2004). It is also important to emphasize the role of the host society, including both Canadian-born individuals and established immigrants, in the adaptation of immigrants. The host society must demonstrate a concerted effort to welcome new immigrants and foster a feeling of ‘place’. A greater focus on immigrant employment (income) and acceptance of the host society, would improve SoP and the overall experience of immigrant adaptation. More research is needed to determine the specific factors that contribute to Charlottetown’s strong SoP, and how this can be implemented in other cities. Further research will employ qualitative methods to further explore immigrant evaluations of SoP.

## Footnotes

1 Presentation on: Immigrants' Quality of Life: Relationship to Adaptation in Three Canadian Cities-.A Focus on Second and Third Tier Cities, Charlottetown-specific Implications. Presented by: Craig Mackie, PEI Association for Newcomers to Canada
